# Community-Directed Vector Control to Accelerate Onchocerciasis Elimination

**DOI:** 10.3390/pathogens13030268

**Published:** 2024-03-21

**Authors:** Benjamin Jacob, Edwin Michael, Thomas R. Unnasch

**Affiliations:** Center for Global Health Infectious Disease Research, College of Public Health, University of South Florida, 3720 Spectrum Blvd, Suite 304, Tampa, FL 33612, USA; bjacob1@usf.edu (B.J.); emichael443@usf.edu (E.M.)

**Keywords:** *Onchocerca volvulus*, *Simulium*, river blindness, remote sensing, trap

## Abstract

Onchocerciasis, or river blindness, has historically been one of the most important causes of blindness worldwide, and a major cause of socio-economic disruption, particularly in sub-Saharan Africa. Its importance as a cause of morbidity and an impediment to economic development in some of the poorest countries in the world motivated the international community to implement several programs to control or eliminate this scourge. Initially, these involved reducing transmission of the causative agent *Onchocerca volvulus* through controlling the vector population. When ivermectin was found to be a very effective drug for treating onchocerciasis, the strategy shifted to mass drug administration (MDA) of endemic communities. In some countries, both vector control and ivermectin MDA have been used together. However, traditional vector control methods involve treating rivers in which the black fly vectors breed with insecticides, a process which is expensive, requires trained personnel to administer, and can be ecologically harmful. In this review, we discuss recent research into alternatives to riverine insecticide treatment, which are inexpensive, ecologically less harmful, and can be implemented by the affected communities themselves. These can dramatically reduce vector densities and, when combined with ivermectin MDA, can accelerate the time to elimination when compared to MDA alone.

## 1. Introduction

Onchocerciasis has historically been one of the most important causes of blindness worldwide [1]. The disease causes infection with the filarial parasite *Onchocerca volvulus*. Historically, onchocerciasis is found in a wide belt of sub-Saharan Africa, spanning from Senegal in the west to Uganda, Sudan, and Ethiopia in the east. It extends from Mali and Sudan in the north of Africa to the Democratic Republic of Congo and Malawi in the south. It is also found in isolated foci in Yemen, as well as in Latin America [2].

*O. volvulus* is an obligate parasite of humans, and it is transmitted by blackflies of the genus *Simulium*. In Africa, the major vectors are members of the *Simulium damnosum sensu lato* complex [3], although other species of *Simulium* (particularly *Simulium neavei* in some foci of East Africa) can also serve as a vector [4]. One major feature shared by the *Simulium* vectors of *O. volvulus* is that the female flies lay their eggs and the larvae develop in well oxygenated, clean, fast-flowing water. The vectors thus localize near the rivers, particularly in and around river rapids. This has led to the colloquial name of “river blindness” for the disease. 

Given there are two organisms involved in maintaining the *O. volvulus* lifecycle (humans and the black fly vector), there are two places where one might attack the parasite to stop transmission. One can try to attack the parasite in humans (through drug treatment) or one can attack it in the vector (through vector control). Until the 1980s, there were no safe and effective drugs that could be used to treat *O. volvulus* in the human population. However, in 1985, ivermectin was shown to be a potent microfilaricide against *O. volvulus* [5]. Early studies also demonstrated that mass treatment of an afflicted population with ivermectin could reduce transmission of the parasite [6,7]. Based upon the dramatic effect of ivermectin on *O. volvulus* and its ability to reduce or eliminate the symptoms of the infection in afflicted individuals, Merck, the manufacturer of ivermectin, announced that they would provide the drug free of charge for the treatment of onchocerciasis, “as much as needed for as long as needed” [8]. As a result of this generous donation, several large international programs were begun to either control or eliminate onchocerciasis, employing a strategy of mass drug administration (MDA) of ivermectin to the afflicted communities. Most notably, these included the African Programme for Onchocerciasis Control (APOC) in Africa and the Onchocerciasis Elimination Program of the Americas (OEPA). OEPA, employing a strategy of semi-annual distribution of ivermectin with high coverage rates, has succeeded in eliminating onchocerciasis in four of the six former countries in Latin America (Colombia, Guatemala, Ecuador, and Mexico), and has interrupted transmission in all but two foci in the region [9]. In Africa, studies conducted in Mali, Senegal [10] and Nigeria [11] have suggested that long-term annual community-directed treatment with ivermectin (CDTI) has succeeded in eliminating onchocerciasis from some isolated foci in West Africa. These successes have resulted in a change in strategic focus in Africa, from a goal of disease control of onchocerciasis to a goal of complete elimination. This goal was enshrined in the London Declaration on Neglected Tropical Diseases in 2010, where the international community set a goal of eliminating onchocerciasis from Africa by 2020 [12].

Despite the successes documented in both Africa and in Latin America, ivermectin alone is not likely to be a panacea. Ivermectin, although very effective at killing the microfilaria of *O. volvulus* (the first-stage larvae transmitted to the black fly vector), its effect on the adult stages of the parasite is limited. This means that repeated MDA rounds are necessary to suppress transmission long enough to allow the adult stages in the humans to die off or become infertile. Additionally, it is impossible to treat every eligible individual in every community in every treatment round. This means that there will always be a low number of adult parasites in the community, who could then re-start transmission once drug pressure is lifted. The actual number of adult parasites necessary to re-start transmission is dependent on the amount of contact that people have with the vector, or the vector biting density. The higher the biting density, the harder it will be to achieve elimination with ivermectin MDA alone. Indeed, in many areas of Africa where vector populations are high, models predict that ivermectin MDA alone will probably not be sufficient to interrupt transmission. For example, studies using a stochastic model indicate that in savanna areas of Africa where the biting rate exceeds roughly 5000 bites/year (which includes many areas in the savanna of sub-Saharan Africa), complete elimination of *O. volvulus* using MDA alone may not be possible [13]. These predictions are supported by studies in Cameroon and Uganda, where it was demonstrated that transmission of *O. volvulus* continued despite 15 and 18 years of annual ivermectin MDA, respectively [14,15]. Thus, in order to attain the goal of eliminating onchocerciasis from Africa, it may be necessary to supplement ivermectin MDA with other interventions. 

Vector control as a tactic to combat onchocerciasis in Africa has a long history. The use of larvicides to eliminate adult flies and to block transmission of *Onchocerca volvulus* was first implemented in Kenya from1946 to 1955 [16] using DDT to eliminate populations of the local vector *Simulium neavei* in the six Kenyan foci of onchocerciasis. Elimination of the vector was successful in interrupting transmission in Kenya, and follow-up studies confirmed that the parasite had been eliminated from these foci [17].

The Kenyan success of targeting the vector population was used as a model for the first international onchocerciasis control program in Africa. The Onchocerciasis Control Programme of West Africa, or OCP, was a large-scale, vertically integrated program whose aim was to eliminate blinding onchocerciasis as a public health problem throughout eleven countries in West Africa where the disease was a significant public health problem. The OCP began operations in 1975 before the advent of ivermectin. It thus relied primarily upon a strategy of vector control, i.e., aerial spraying of *Simulium damnosum sensu lato* larval breeding sites. The OCP was a disease control program and not an elimination program; thus, while elimination of the parasite did not occur over the entire area under control by the OCP, elimination of severe ocular disease was achieved in its core transmission areas after 14 years of vector control [18]. A great deal of public health value was accomplished by this landmark effort throughout the region. Skin disease was significantly reduced, more than 200,000 cases of blindness were prevented, and the size and distribution of the *O. volvulus* population in the region were substantially decreased [19]. The OCP completed its operations in 2002. 

More recently, Uganda has demonstrated the power of utilizing a combination of vector control and ivermectin MDA. The first evidence for this came from western Uganda, where it was found that combining vector control (larviciding small streams supporting populations of *Simulium neavei*) with ivermectin MDA resulted in a rapid decline in the transmission and in prevalence of infection in the human population [20]. In 2007, this observation was incorporated into the strategic plan of the newly formed Uganda Onchocerciasis Elimination Program (UOEP). Since beginning operations in 2007, the UOEP has used a strategy that combines the use of vector control (local larviciding of vector breeding sites) with semi-annual MDA. This has resulted in the apparent interruption of transmission of onchocerciasis in 15 of the 17 foci in Uganda to date [21]. These data support the hypothesis that vector control, used in combination with ivermectin MDA, is a powerful strategy to eliminate onchocerciasis. 

Vector control has proved its success in controlling and in some cases eliminating onchocerciasis. However, classical methods of vector control using insecticides have some significant drawbacks. Adding insecticides to rivers, which are often used by the local people as water sources for drinking and bathing, can have consequences for both the health of the local population and the riverine ecology. Second, classical vector control requires the use of insecticides that can be expensive and generally must be imported. Finally, to be effective, they must be applied by trained technicians who can calculate both the flow volume of the river and the dosage of insecticide necessary. This skill set is not commonly found in the villages of rural Africa. If one is going to apply vector control generally across Africa, cheaper, safer, and less technically complex methods are needed. 

In this review, we present some recent advances in the development of community-directed methods for the control of the vectors of *O. volvulus*. First, we discuss the development of traps that can be used to reduce biting of the vectors, and slash and clear methods to remove the substrates that the blackfly larvae need to attach to in order to complete their development. Then we discuss advances in ways to identify the breeding sites of the *Simulium* blackflies, which is a necessary step in developing a vector control strategy. Finally, we discuss results of modeling studies that attempt to assess how the community-directed methods may be useful in accelerating the push to elimination.

## 2. The Esperanza Window Trap (EWT)

An important metric for verifying the elimination of onchocerciasis from a country is demonstrating that transmission has been suppressed prior to stopping MDA and then ensuring it has not re-started after MDA has been withdrawn for 3–5 years [22]. Demonstrating that transmission has been suppressed and then interrupted requires the collection of large numbers of vector black flies and then screening them for the presence of *O. volvulus* infective-stage larvae. Traditionally, the collection of *Simulium* vector blackflies has been conducted using human landing collectors. However, human landing collections are inefficient and pose the risk of infection to the collectors if the flies are not caught before they start to take a blood meal. For this reason, the use of human landing collections has become ethically questionable, and an alternative method of collecting the vector black flies was required. In 2013, Rodriguez and colleagues reported the development of a trap (the Esperanza Window Trap, or EWT) that could be used to replace human landing collectors for the collection of the Latin American vector *Siumulium ochraceum* [23]. This trap consisted of a 1 m^2^ piece of blue fabric (plastic tarpaulin) attached to a wooden frame and coated with a sticky glue. The trap was baited with carbon dioxide and a chemical lure [23]. Field studies suggested that the EWT collected between 50% and 100% of the number of vectors that were collected by a human landing collector [23]. Since a single individual was capable of maintaining several traps at once, the EWT appeared to be a suitable alternative to human landing collections. Additional studies demonstrated that the EWTs could be operated and maintained successfully by unsupervised community members, making them an economical alternative to human landing collectors [24]. 

**Figure 1:**
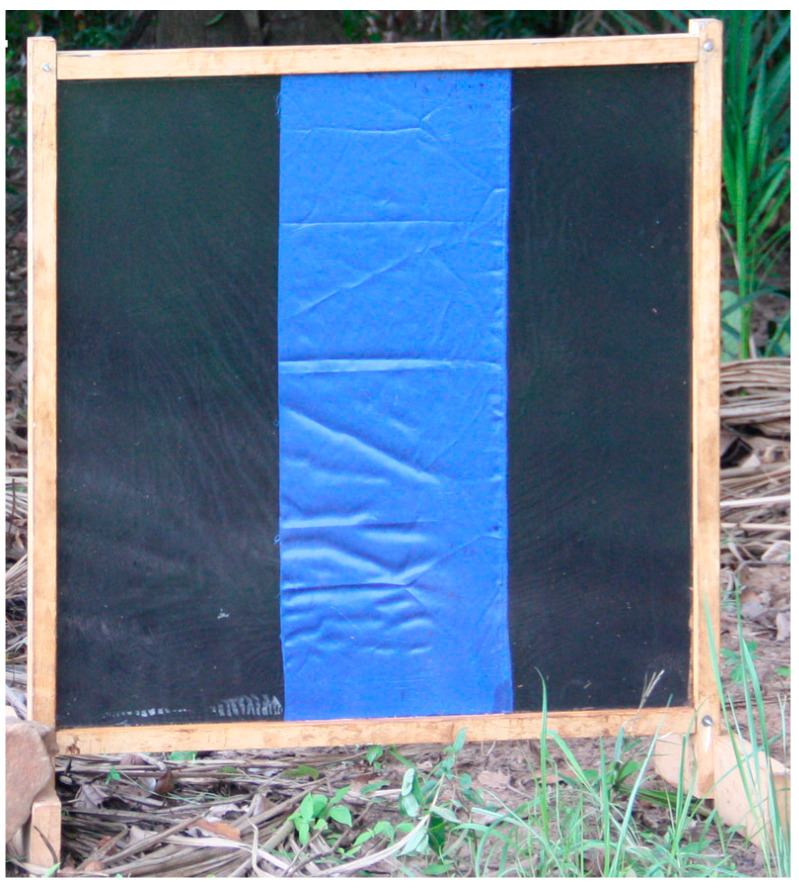
The EWT optimized for Burkina Faso. Figure is adapted from [25].

**Figure 2:**
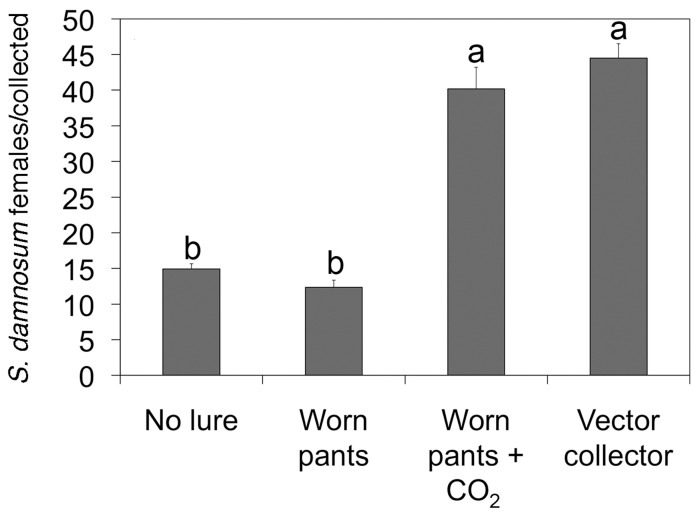
Performance of the optimized EWT versus human landing collections; the daily catch of *S. damnosum s.l.* for different baits used on the EWT was compared to the daily catch from a human landing collector. Letters indicate trails in which the difference between the catch rates was not statistically significant. Figure is adapted from [25].

The EWT design from Mexico was then optimized in Burkina Faso to collect the main vector of *O. volvulus* in Africa, *Simulium damnosum* sensu stricto. In Burkina Faso, it was found that the optimal design consisted of a 1 m^2^ of blue tarpaulin painted with vertical black stripes (Figure 1), and baited with a combination of carbon dioxide and worn pants from a local resident in place of the commercial lure. After optimization, a single trap was found to collect similar numbers of vectors when compared to those collected by a human landing collector (Figure 2). 

**Figure 3:**
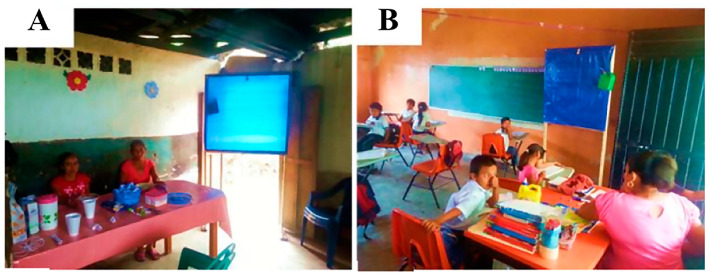
Deployment of EWTs in households and elementary schools. The EWTs were deployed at the kitchen–dining area of the household (**A**) and at the rear of the classroom of the elementary school (**B**). Figure adapted from [26].

**Figure 4:**
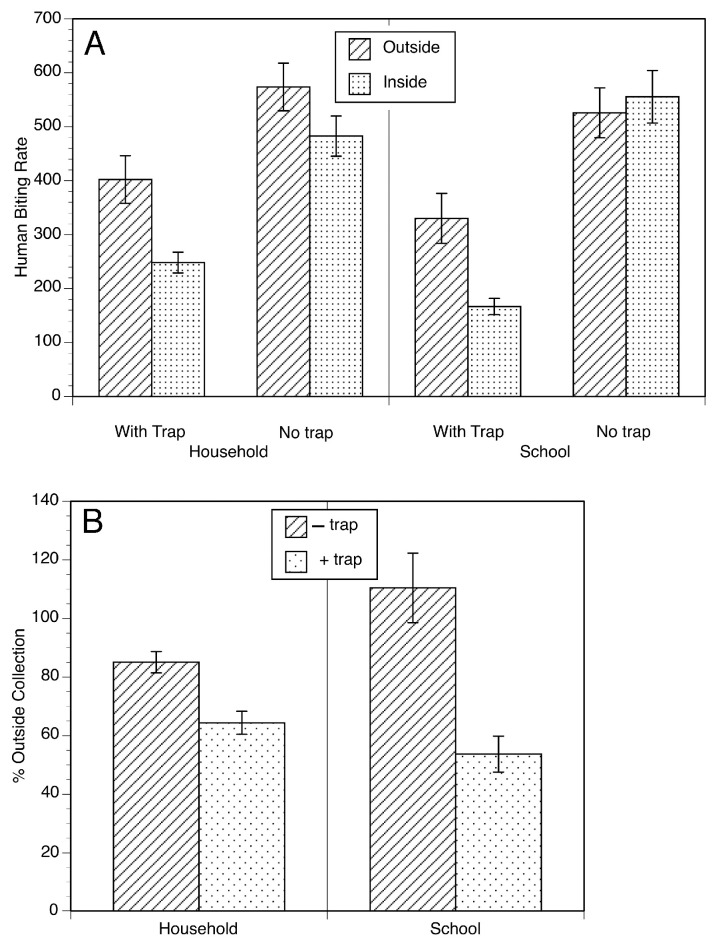
The biting rate of *S. ochraceum s.l.* in household and elementary school in the presence and absence of traps: (**A**) Human biting rate indoors and outdoors in the presence and absence of the EWTs. The human biting rate was calculated as the least square mean of the number of flies per human landing collecting team per day ± SE. (**B**) Indoor biting rate in the presence and absence of the EWTs normalized to the outdoor biting rate. Figure adapted from [26].

During the trials of the EWT in Mexico, it was observed that black flies would be attracted to the people setting and maintaining the traps, but as they got close to the individual and the trap, they diverted and often landed on the trap. This suggested that the EWT might be able to divert host-seeking flies, causing them to stick to the trap, and thereby reducing the biting of the individuals nearby. To test this hypothesis, trials were carried out in households and in a school in Oaxaca, Mexico, to see if placing the EWTs near or in the rooms of the school and homes might reduce the biting rate (Figure 3) [26]. Significant reductions in the biting rate were observed in both household and school locations, with a greater effect being seen in the school setting (Figure 4).

**Figure 5:**
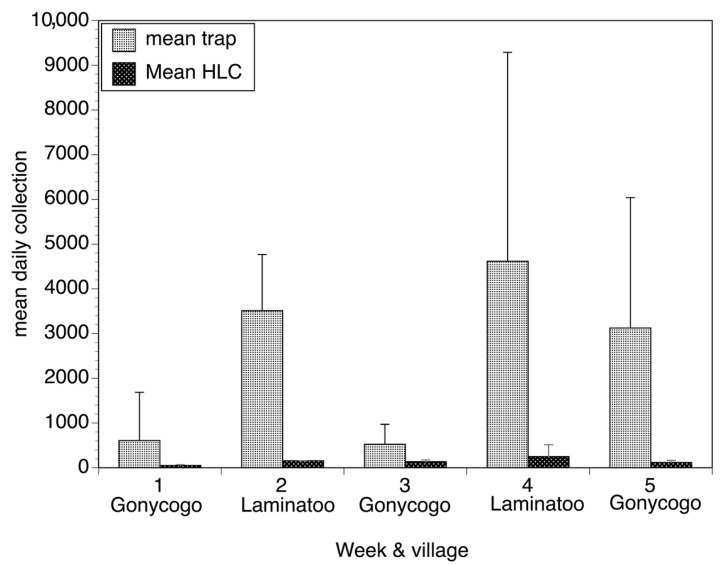
Daily collections obtained by traps and by the human landing collector in Gonycogo and Laminatoo, Uganda. Bars represent the mean daily collection obtained by each of the five individual traps situated around the two fields in each village and the mean collection obtained by the two HLCs situated in each field over the five weeks of the study. Error bars depict the standard deviation surrounding the mean daily collections. Data from each week were calculated from six days of human collection data and 30 days of trap collection data. Figure adapted from [27].

**Figure 6:**
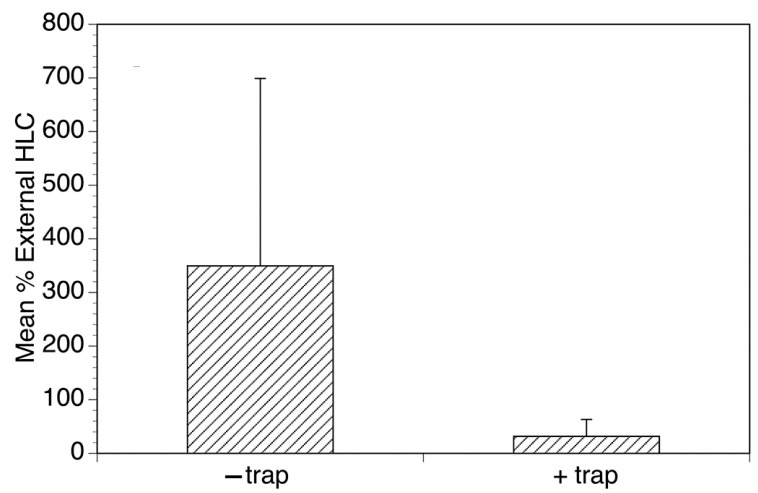
Evaluation of the ability of the optimized EWT to reduce biting in a school setting. Mean collections obtained by collectors in the classrooms normalized to those obtained by the external collector in the presence and absence of the traps. Bars represent the mean percentage of the collections obtained by the collectors relative to those obtained by the external collector in the presence and absence of the traps, and error bars represent the standard deviations of the normalized counts. Figure adapted from [27].

**Figure 7:**
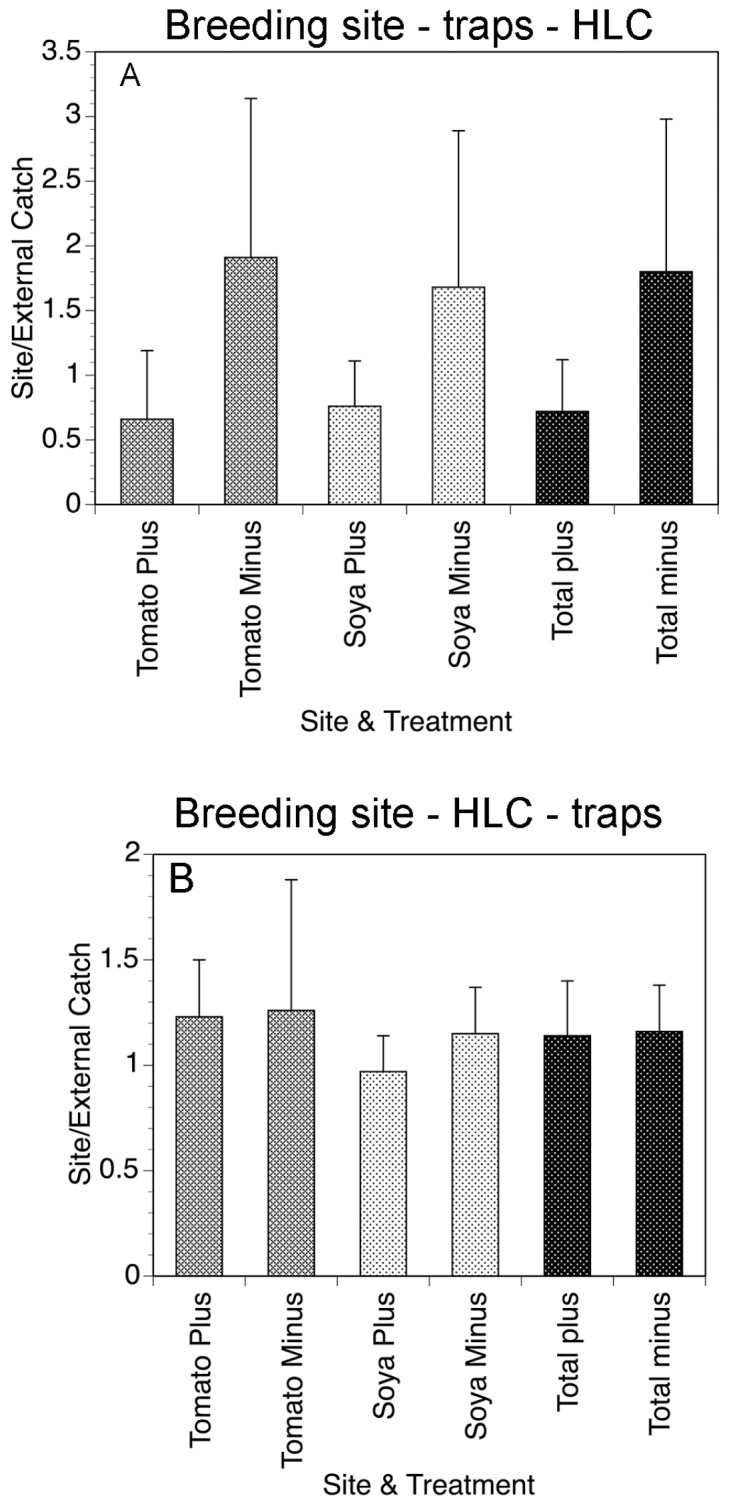
(**A**) Weekly fly collections in tomato and soya fields of Gonycogo in the presence and absence of the traps. (**B**) Mean weekly collections on the tomato and soya fields of Laminatoo in the presence and absence of the traps. Data are normalized to the number of flies collected by an external collector, to account for variations in the overall fly population. Bars represent the standard deviations of the means. Figure adapted from [27].

Concurrent with the studies in Mexico, optimization studies of the EWT continued in northern Uganda. These resulted in a version of the trap (1 m^2^ black-and-blue-striped tarpaulin baited with yeast producing carbon dioxide, and dirty socks) that significantly outperformed a human landing collector (Figure 5) [27]. The Uganda optimized EWT was tested to see if it could reduce biting in an open-air classroom and in an agricultural setting, using a protocol similar to that used in Mexico. The EWTs dramatically reduced biting in the classroom, similar to what was seen in Mexico (Figure 6) [27]. Some reductions were also seen when the traps were deployed in the field, though this was dependent on the position of the trap relative to the fly breeding site and the workers in the field (Figure 7) [27].

## 3. Slash and Clear

**Figure 8:**
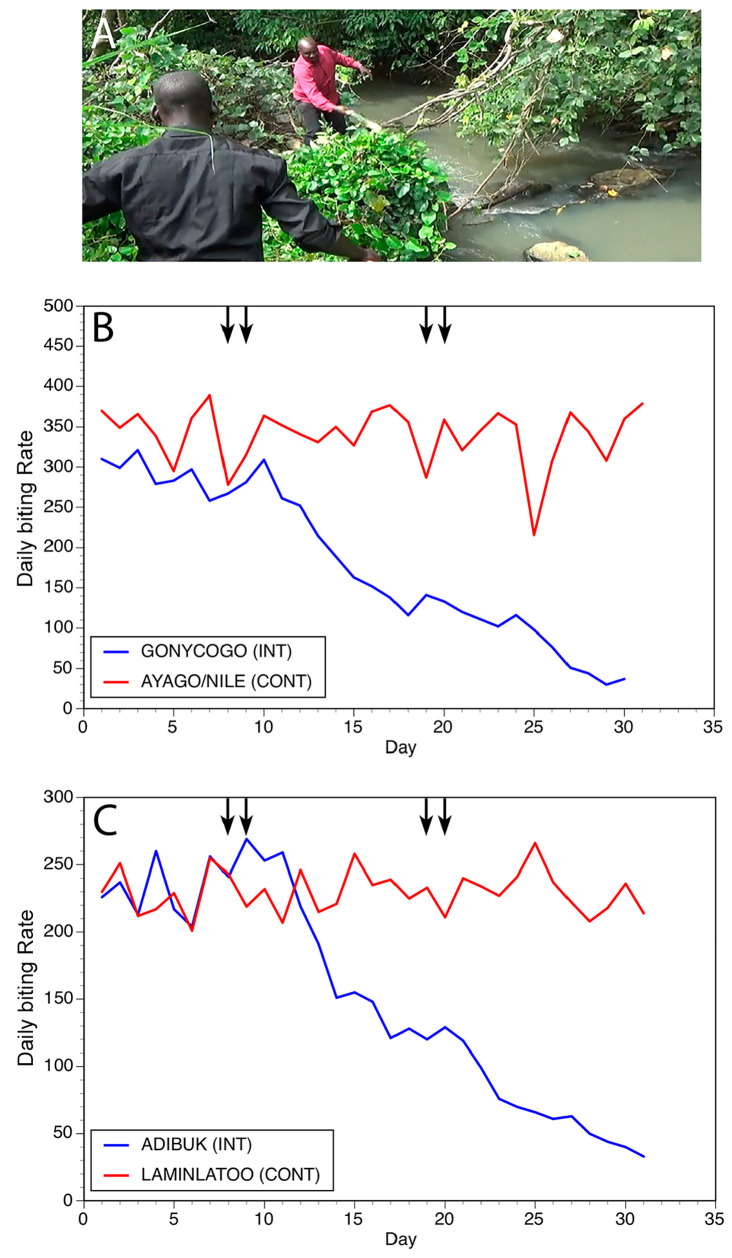
Results of slash and clear interventions carried out on the Ayago river: (**A**) Vegetation clearance at a typical breeding site on the Ayago. (**B**) Daily biting rates before and following interventions at one matched pair of communities. (**C**) Daily biting rates before and following interventions at a second matched pair of communities. In Panels (**B**,**C**), INT = intervention community and CONT = control community. Vertical arrows indicate days during which slash and clear activities were carried out. Figure originally published in [28].

**Figure 9:**
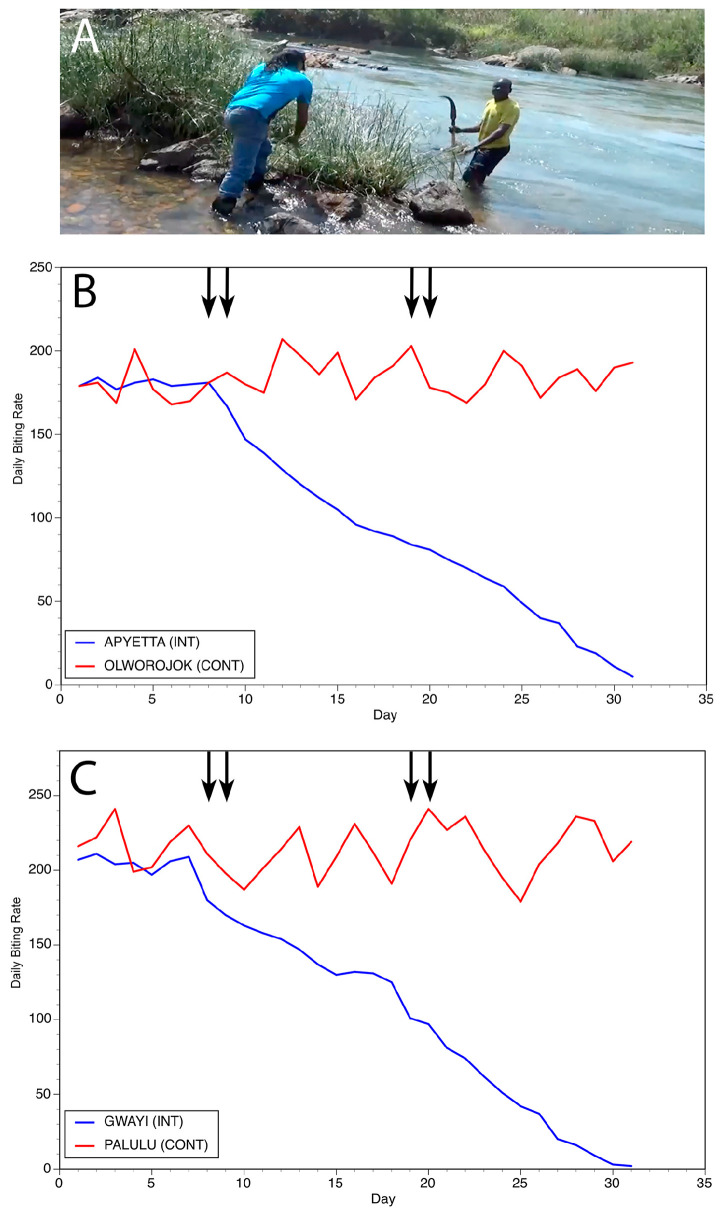
Results of slash and clear interventions carried out on the Aswa river: (**A**) Vegetation clearance at a typical breeding site on the Aswa. (**B**) Daily biting rates before and following interventions at the first matched pair of communities. (**C**) Daily biting rates before and following interventions at the second matched pair of communities. In Panels (**B**,**C**), INT = intervention community and CONT = control community. Vertical arrows indicate days during which slash and clear activities were carried out. Figure originally published in [28].

As discussed above, *Simulium* black flies lay their eggs in clean, fast-flowing water. When the eggs hatch, the larvae remain in the fast-flowing water by attaching to immobile substrates in the stream. The trailing vegetation found along the river banks bordering the rapids is the favored substrate for the larvae. We hypothesized that trimming the trailing vegetation to which the larvae attach along the breeding sites would remove the substrates that the larvae needed to attach to and develop, thereby reducing the fly population. To test this hypothesis, two trials were carried out in Northern Uganda [28]. One trial was carried out on the Ayago river, a small river averaging 2m wide. The second trial was carried out on the Aswa river, a larger river measuring 11m wide. In both trials, matched pairs of control and intervention villages were chosen for the study. Fly collections were carried out in all villages for one week to establish baseline biting rates. Following the pre-intervention period, members of the intervention community were enlisted to trim the trailing vegetation at breeding sites located within 1 km of the community. The vegetation clearing process was repeated two weeks later in the intervention communities. No interventions were carried out in the control villages. Fly biting rates were monitored in all villages for a total of 30 days. In both trials, biting rates began to decline 16 days after the first treatment in the intervention villages. In the villages along the Ayago river, biting was reduced by 89% at day 30 when compared to the initial biting rates, while in the villages along the Aswa, biting was reduced by 99% (Figure 8 and Figure 9). Given how the substrate removal was conducted, the process was named “slash and clear” [28].

Additional studies were then carried out to determine the optimal distance from a community where slash and clear was needed to be carried out to maximize its effect and how often it needed to be performed to maintain the effect. The protocol used in these studies was similar to that used on the Ayago and Aswa rivers, except that only one slash intervention was carried out. When slashing was limited to 1 km from a community in these studies, biting was reduced by an average of 74% [29]. When slashing was extended to a 2 km radius, an average reduction of 95% in the biting rate was observed [29]. Extending the slash out to 3 km from the communities did not result in any further reduction in biting rates [29]. 

Long-term studies were then carried out to determine the optimum time of the year to conduct slashing operations [29]. It was found that two slash operations, conducted at the beginning and end of the rainy seasons (June and November), were maximally effective in maintaining the biting rate at near zero until the start of the following rainy season [29].

Other studies have also shown slash and clear to be an effective form of vector control [30]. In studies carried out at the Maridi dam spillway in the Republic of South Sudan, biting rates were decreased by >90% for six months after a round of slash and clear. The biting rate remained suppressed, rebounding to less than 50% of the initial biting rate one year after treatment [30].

## 4. Breeding Site Identification

For slash and clear to be implemented successfully, it is necessary to identify all breeding sites within 2 km of the at-risk communities. As mentioned above, in the case of the *Simulium* vectors of *O. volvulus*, the blackflies only lay their eggs in fast-flowing, clean, well-oxygenated water. This limits the source of the vector to certain locations in the rivers, primarily rapids. Thus, breeding sites can be located relatively easily if trained entomologists can walk along the river banks, checking the trailing vegetation in the fast-flowing stretches of river for black fly larvae. However, in many places in Africa, it is very difficult to conduct such ground-based prospections. The brush along the rivers is often difficult to penetrate, slowing progress. Additionally, travel by foot along the rivers can be dangerous, as one may encounter animals (e.g., hippos and snakes) that may be a lethal threat. The use of remote methods to locate potential breeding sites can help by limiting the amount of a river that needs to be investigated by foot. Over the past decade, several methods have been developed that use remote sensing (satellite) images to identify potential breeding sites. Three of these have recently been combined into a single iOS interactive application (app). This app uses geo-spatial artificial intelligence [AI] machine-learned [ML] methods to analyze unmanned aerial vehicle [UAV] and/or satellite sensed data to detect areas where larval control for *S damnosum s.l.* can be effectively implemented. The app does this by first identifying areas that are suitable for *S. damnosum s.l.* larvae, which require well-oxygenated sediment-free water. The app then analyzes the potential larval habitats to predict the tactics that will be most effective at eliminating onchocerciasis in the adjacent communities (i.e., S&C plus MDA, S&C combined with EWT deployment plus MDA, and EWT deployment plus MDA or MDA alone). 

The first algorithm employed in the app was previously described in Jacob et al. [31]. It employs the ENVI software V5.7 package (Exelis Visual Information Solutions, Boulder, CO, USA) to derive spectral reflectance estimates of field-validated *S. damnosum s.l.* breeding sites, using a geometric-optical model. A land cover pixel signature is used to classify imagery according to how closely a pixel region matches the signature of the field-validated sites. This is then combined with information from the Orfeo-Toolbox, which has a Spectral Angle Classification algorithm, based on work by Du and co-workers [32]. The algorithm then employs a set of reference pixels to compute a spectral measure to find the pixel regions similar to the signature found at the field-validated sites. 

The second algorithm employs an unsupervised clustering Iterative Self-Organizing Data Analysis Technique (ISODATA) as implemented in the ERDAS Imagine v.8.7 software package (ERDAS, Inc., Atlanta, GA, USA). As described in Jacob et al. [31], these iterative techniques automatically group signature *S. damnosum s.l.* pixels of similar spectral features into unique clusters.

**Figure 10:**
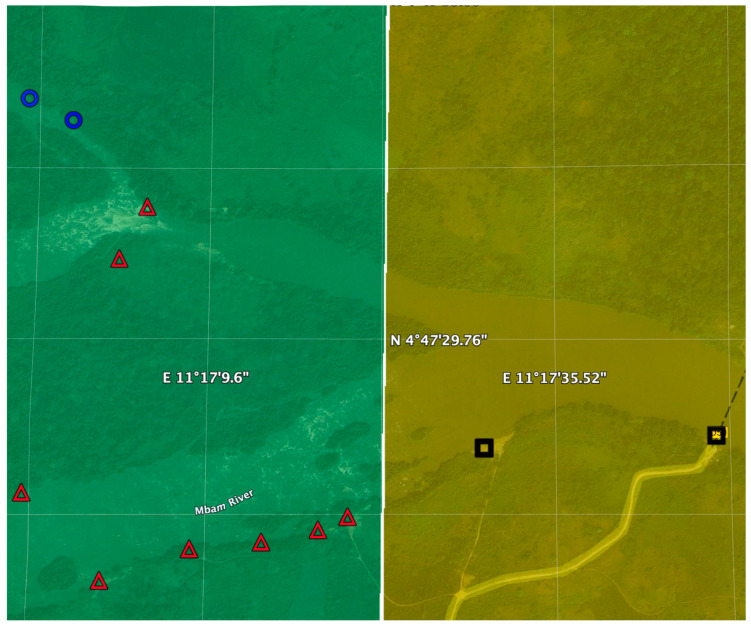
Identification of potential breeding sites for vector control intervention: Predicted locations for implementing S&C, EWT or MDA alone along the Sanga River basin in Cameroon using the model described in the text. Gridlines correspond to 1000 m squares. The watercourses highlighted in yellow contain predicted *S. damnosum* s.l. habitat. Grids in orange are predicted to have a high probability of high vector biting while those in green are predicted to have a lower density of vector biting. Blue circles correspond to communities where MDA alone is predicted to be the optimal elimination strategy. Red triangles correspond to communities where EWT traps and MDA are optimal, while black squares correspond to communities where MDA plus slash and clear are recommended. Figure adapted from [31].

The third algorithm is an application of a digital elevation map (DEM) overlay on the collected high-resolution satellite imagery [33]. The DEM identifies where steep fast-flowing sections of river could form under certain conditions. Combining an overlay of a DEM and the clustering and spectral analysis results of the previous two algorithms allows one to predict which of the larval control strategies will be most appropriate at the different potential breeding sites (Figure 10).

## 5. Modeling the Impact of Slash and Clear in Accelerating Elimination of Transmission

**Figure 11:**
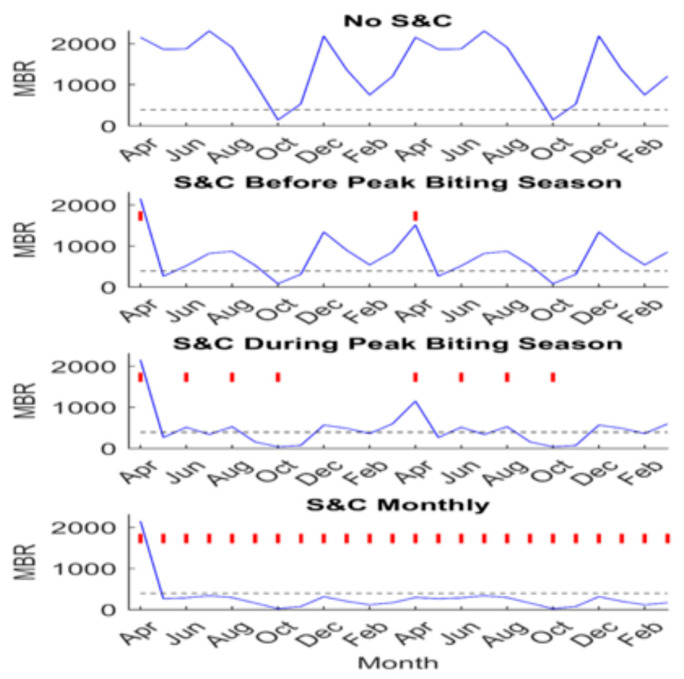
Impact of “slash and clear” on MBR for different intervention schedules in Masaloa, Uganda. Two years of implementing S&C along with annual MDA are shown, with vertical red lines indicating the months where vegetation was cleared. The blue line depicts the median MBR prediction throughout the intervention period and the horizontal black dashed line represents the median TBR for Masaloa. From [34].

Mathematical models of *O. volvulus* transmission have been used to evaluate the role that slash and clear (S&C) can play in accelerating the achievement of elimination of transmission (EoT), based on various elimination thresholds and across locations varying in baseline infection endemicities [34]. Various S&C scenarios (in combination with annual MDA at 80% population coverage) have been analyzed using these models. These scenarios ranged from using MDA alone to implementing different frequencies of S&C in conjunction with annual MDA. The different frequencies included modeling the administration of S&C during the first month of the vector biting season, every other month during the biting season, and at monthly intervals throughout the year. These different strategic approaches were modeled and compared for their effectiveness in reaching WHO thresholds for the elimination of onchocerciasis in four Ugandan communities that differed in baseline mf prevalence, ranging from 24% to 100%. The simulations were carried out using a seasonally driven deterministic population dynamics model of onchocerciasis fit or calibrated to locality-specific mf age-prevalence and different intensities of vector biting (as measured by the seasonal monthly biting rate [MBR]), using an ensemble-based Bayesian Melding (BM) approach as described fully in Michael et al. [35] and Smith et al. [34]. The results of these simulations are reproduced in Table 1 and Figure 11, which clearly underscore how supplementing MDA with vegetation-clearing activities can present a potent strategy for accelerating the achievement of onchocerciasis elimination, compared to relying on annual MDA alone. Indeed, these results indicate that implementation of S&C can potentially save more than 10 years of interventions compared to relying on annual MDA alone if mf thresholds (irrespective of whether these are model-predicted or WHO-defined) are used as elimination targets, and, notably, the savings could increase to more than 20 years if the corresponding ATP thresholds are used (Table 1). This finding shows that the addition of vector control does not directly result in significant reductions in the community mf prevalence. Instead, it raises the mf breakpoint value, making it easier to reach through MDA. However, ATP thresholds are reached markedly earlier than mf breakpoints (Table 1). This suggests that in locations where vector migration is not a concern, targets based on indicators of infection in the vector population (ATP) could be significantly more sensitive for detecting the eventual interruption of transmission than the corresponding indicators in the human. This is because of the significant lag associated with the decay in mf prevalence, even in the absence of ongoing transmission from the vector, owing to the significantly longer life-spans of adult *O. volvulus* worms when compared to the life-span of *Simulium*. However, in settings where the in-migration of black flies is likely, MDA will still be important for reducing the intensity of the remaining mf infections in order to achieve the permanent reduction in transmission; here, adding S&C to continuing MDA interventions will still significantly reduce the number of years required for this extended drug intervention (Table 1). Finally, it is also apparent that the impact of adding S&C to MDA to reduce the number of years of interventions required to achieve elimination thresholds is greater for settings with lower pre-control prevalence (Table 1).

## 6. Research Challenges Remaining

The studies reviewed above demonstrate that community-based vector control measures can accelerate the process towards elimination and help protect areas where elimination has been achieved from recrudescence. However, challenges remain in widely implementing these techniques across Africa. In the case of the traps, it is unlikely that the existing versions of the traps will perform optimally everywhere in Africa, as the particular species of black vector varies depending on location and habitat. Indeed, studies deploying the EWT design optimized for Uganda in Tanzania found that the traps were not very effective in collecting the local vector species [38]. It is therefore likely that the trap designs and baits will need to be optimized for the local vector species present in a given area.

Similarly, it is likely that slash and clear will not be generally applicable. In some cases, breeding sites are located on large rivers, where entry into the river to clear the trailing vegetation will be too dangerous. In contrast, in the heavily forested habitats of central and west Africa, breeding sites may be located on the many small streams in the forest, making them so numerous that it will be impossible to locate and clear all of them near the afflicted communities. In these situations, alternatives to slash and clear may need to be found.

Finally, in order for the community-directed methods to be effective in the long term, they will need to be sustained by the communities. Sustainability may be facilitated by the fact that, apart from their role as a vector, black flies represent a significant nuisance to the communities where they are present. The bites can be painful, numerous, and frequent enough to disrupt the resident’s daily activities. It is possible that the nuisance caused by the flies may be sufficient incentive for the afflicted communities to continue vector control in the absence of external support. Further studies to determine if this is the case and how to incentivize the communities to sustain these activities are needed. 

## Figures and Tables

**Table 1:** Number of years of interventions required to reach skin microfilaria (mf) prevalence and ATP transmission thresholds.

Village (Baseline mf Prevalence (%), Model-Predicted mf Breakpoints at ABR and TBR (%))	Skin mf Prevalence Threshold				ATP Threshold			
	No S&C	S&C before Peak Biting Season	S&C during Peak Biting Season	S&C Monthly	No S&C	S&C before Peak Biting Season	S&C during Peak Biting Season	S&C Monthly
	**Model-predicted thresholds**							
Palaure Pacunaci (100, 0.7, 0.08)	34 (24–49)	26 (16–45)	25 (16–43)	24 (16–41)	28 (16–50)	10 (2–23)	8 (1–18)	4 (1–12)
Masaloa (76, 0.9, 0.1)	31 (19–49)	19 (11–33)	19 (10–31)	18 (10–29)	20 (10–34)	7 (1–17)	5 (1–14)	1 (1–9)
Nyimanji (58, 0.8, 0.1)	30 (18–47)	19 (10–34)	19 (10–33)	18 (10–32)	18 (8–33)	7 (1–18)	5 (1–14)	1 (1–9)
Olimbuni/Aroga (24, 0.5, 0.1)	28 (15–46)	20 (9–38)	19 (9–36)	19 (9–34)	17 (8–32)	8 (1–18)	5 (1–14)	1 (1–9)
	**WHO thresholds**							
Palaure Pacunaci (100, 0.7, 0.08)	25 (15–45)	24 (15–41)	23 (15–40)	22 (14–37)	19 (9–45)	16 (7–32)	13 (4–26)	9 (1–19)
Masaloa (76, 0.9, 0.1)	20 (11–34)	19 (10–32)	19 (10–31)	18 (10–29)	13 (4–25)	10 (1–21)	8 (1–18)	1 (1–12)
Nyimanji (58, 0.8, 0.1)	19 (9–34)	18 (9–31)	17 (9–30)	17 (9–29)	11 (2–24)	8 (1–20)	6 (1–16)	1 (1–10)
Olimbuni/Aroga (24, 0.5, 0.1)	15 (5–30)	14 (5–28)	14 (5–26)	14 (5–26)	10 (1–22)	7 (1–17)	4 (1–13)	1 (1–8)

The number of years of required interventions to reach mf and ATP thresholds are reported as median predictions with their 95% confidence intervals. All S&C scenarios are in combination with annual MDA at 80% population coverage. Results for both the model-predicted site-specific thresholds (representing 95% elimination probability (see [22,35,36,37]) and the global WHO thresholds (mf prevalence = 1%, ATP = 20) are shown.

## Data Availability

No new data are included in this review.
